# Supplementary material for: Symmetry-adapted Markov state models of closing, opening, and desensitizing in α 7 nicotinic acetylcholine receptors
Source: Nat Commun. 2024 Oct 18;15:9022. doi: 10.1038/s41467-024-53170-z (PMC11489734; doi:10.1038/s41467-024-53170-z)
Supplement: Supplementary file 1 — Supplementary Information [file 41467_2024_53170_MOESM1_ESM.pdf]

Supplementary Information

Yuxuan Zhuang, Rebecca J. Howard, and Erik Lindahl

Supplementary Table 1 Summary of simulation details for two systems. This includes the simulation length (T), the number of independent simulations, the initial box size, the total count of atoms and water molecules, the salt concentration, and the quantity and types of lipids present in the simulated system.

System	T (ns)	# seeds	box (nm)	# atoms	# water	NaCl	# lipid
apo	800-1400	195	12 x 12 x 17	247000	55500	0.15M	400 POPC
CHOL	1200-2240	150	12 x 12 x 18	255000	55500	0.15M	300 POPC + 150 CHOL

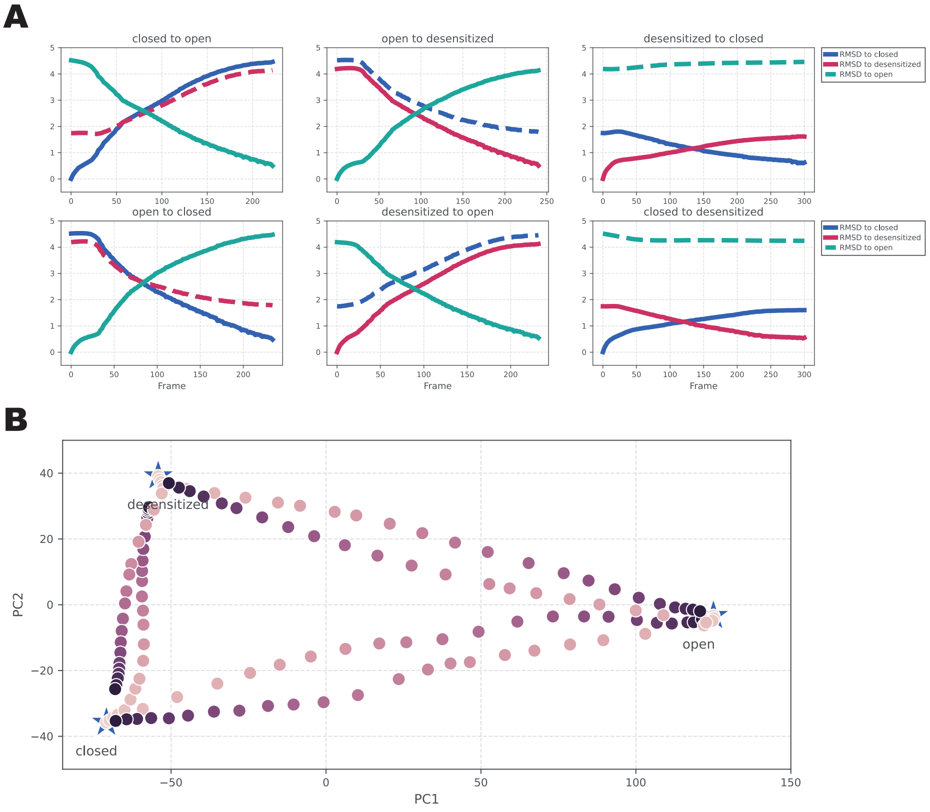

Supplementary Figure 1 Interpolation analysis of Climber trajectories. (**A**) Root mean square deviation (RMSD) of each interpolation from the three structural models. (**B**) Projection of selected seeds along each interpolation onto the principal components generated from the structural models.

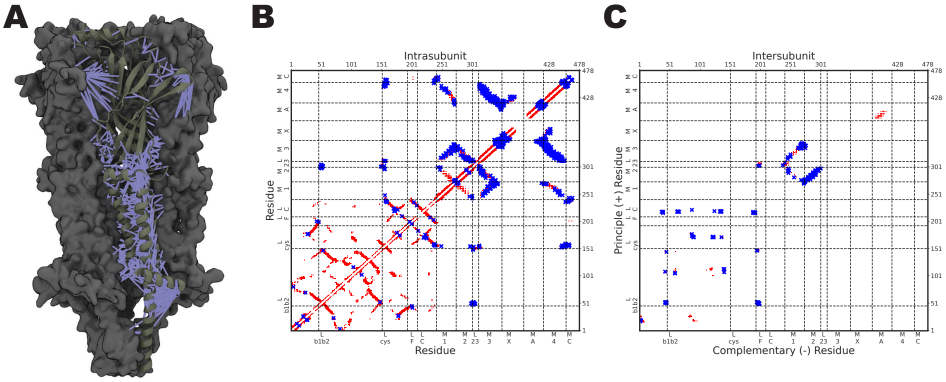

Supplementary Figure 2 Feature selection and contact analysis. (**A**) The selected features for the analysis are highlighted in blue, representing interatomic distances. (**B**) Intrasubunit C_α contacts are shown in red, while the selected contacts are highlighted in blue. (**C**) Intersubunit C_α contacts are shown in red, and selected contacts are highlighted in blue.

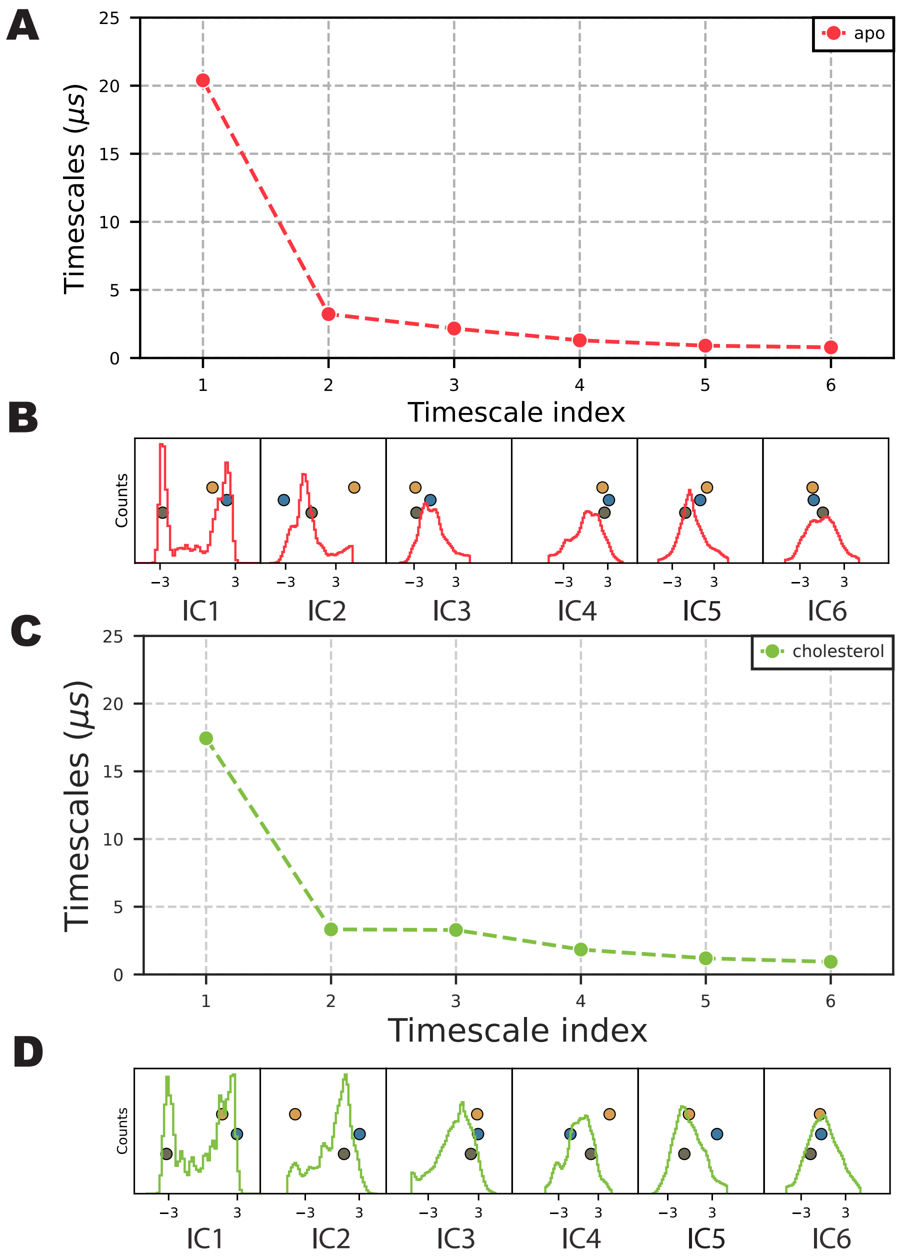

Supplementary Figure 3 Implied timescale of each independent component. (**A, C**) Implied timescale of each independent component for the apo and CHOL systems. (**B, D**) Histogram of each independent component for the apo and CHOL system. Structural models are projected onto the plot (grey: closed; blue: open; yellow: desensitized).

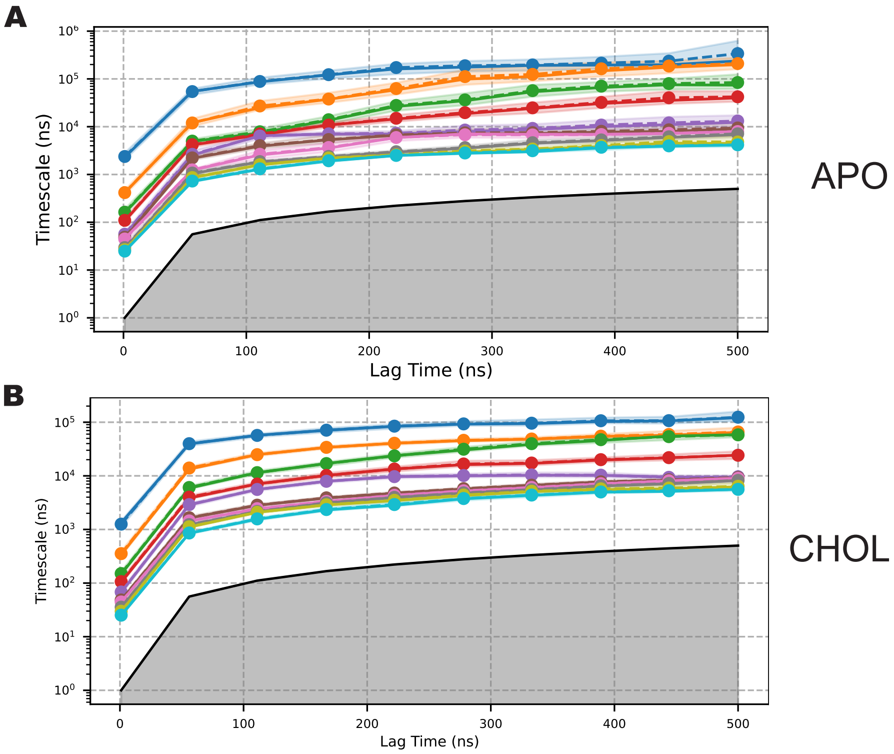

Supplementary Figure 4 Assessment of convergence of the MSMs with implied timescales. (**A, B**) Implied timescales with MSMs estimated with different lag times for apo and CHOL systems.

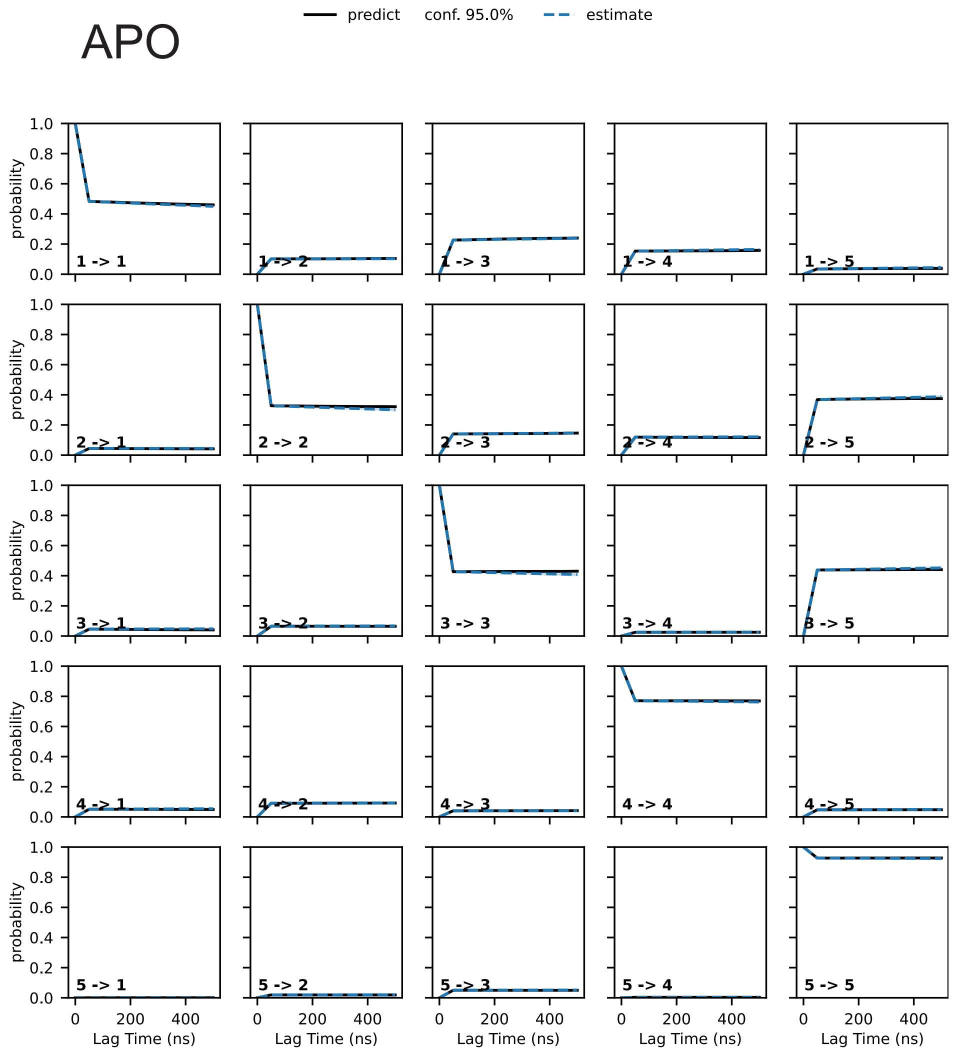

Supplementary Figure 5 Assessment of convergence of the APO MSM with Chapman-Kolmogorov tests. Chapman-Kolmogorov tests with five states for the apo system.

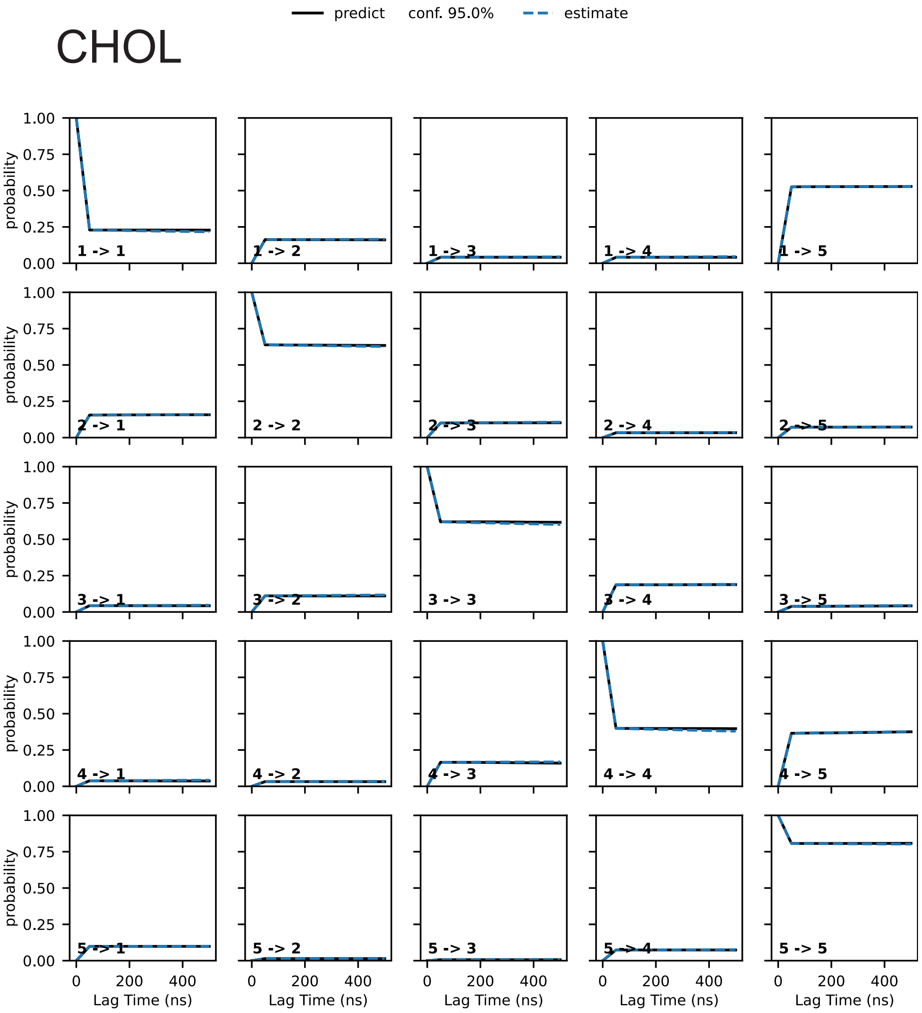

Supplementary Figure 6 Assessment of convergence of the CHOL MSM with Chapman-Kolmogorov tests. Chapman-Kolmogorov tests with five states for the CHOL system.

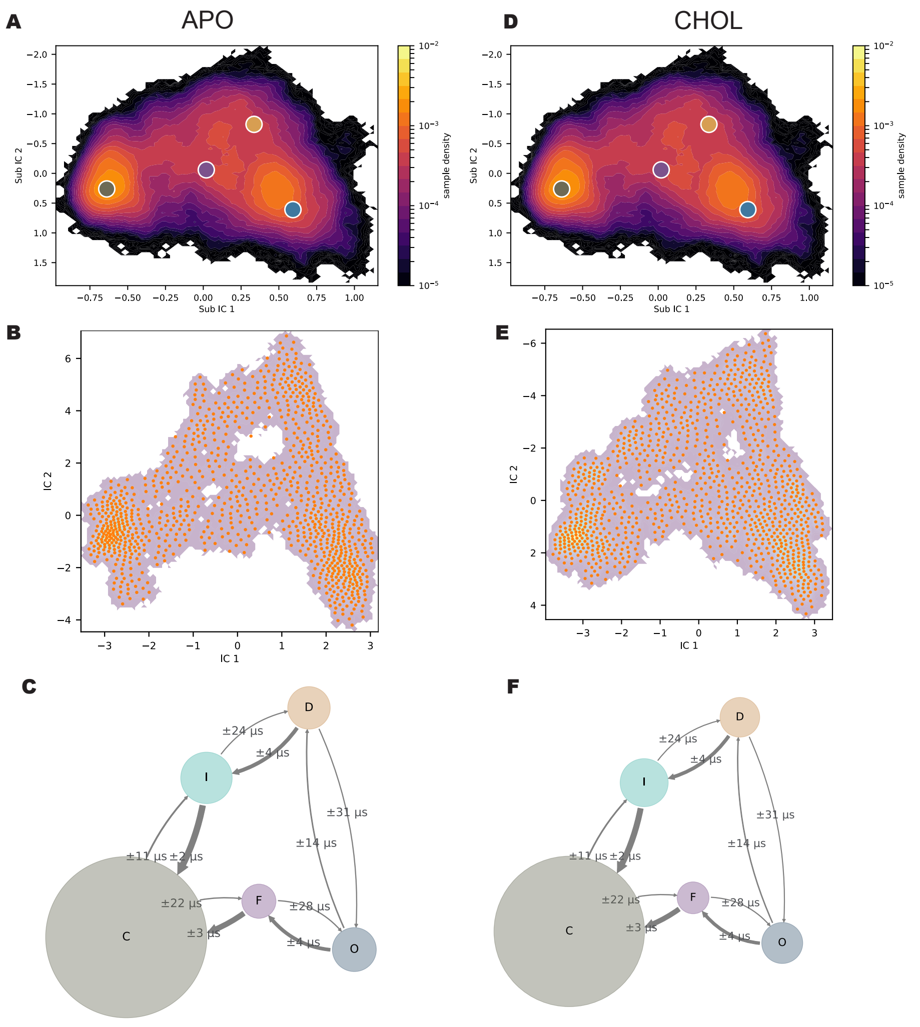

Supplementary Figure 7 Microstates in MSMs and kinetic error estimation. (**A D**) Projection of the four structural models onto subIC1-IC2 coordinate density map for apo and CHOL systems. (**B E**) 1000 microstates assigned with k-mean clustering for apo and CHOL systems. (**C F**) The standard deviation for each mean first passage time for apo and CHOL systems based on Bayesian MSM (100 samples).

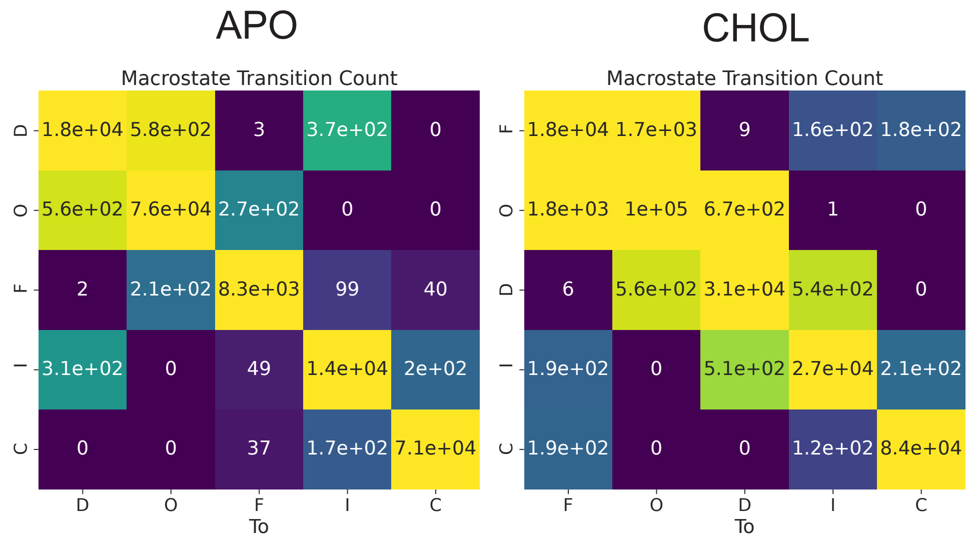

Supplementary Figure 8 Transition counts in MSMs. Number of transitions between each macrostate for apo and CHOL systems, colored indigo–yellow with increasing number of transitions.

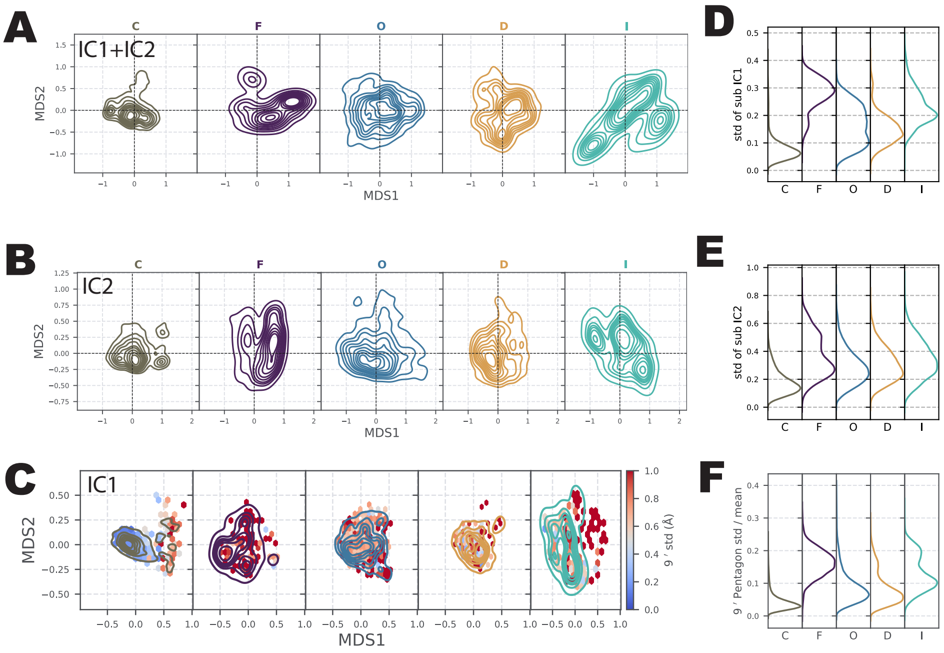

Supplementary Figure 9 Standard deviation and MDS of other ICs.(**A**) Projection of sub independent components (IC1+IC2) onto MDS space. (**B**) Projection of subIC2 onto MDS space. (**C**) Projection of subIC1 onto MDS space. The histogram of the standard deviation of the five edges on the 9′ C_α pentagon is plotted as a heatmap. (**D,E,F**) Distribution of the standard deviation of subIC1, IC2, and normalized 9′ C_α pentagon edge lengths in each macrostate. All distributions are weighted by the MSM weights.

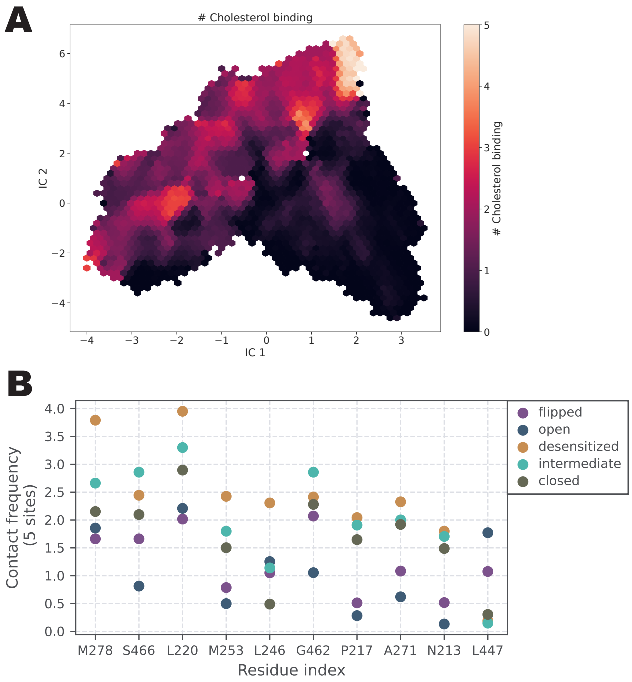

Supplementary Figure 10 Differential binding of cholesterol in each macrostate (**A**) Number of cholesterol molecules bound to the intersubunit binding site mapped onto IC1-IC2 coordinates. (**B**) Top 10 residues interacting with cholesterol in each macrostate, ordered by the greatest variance across five states.

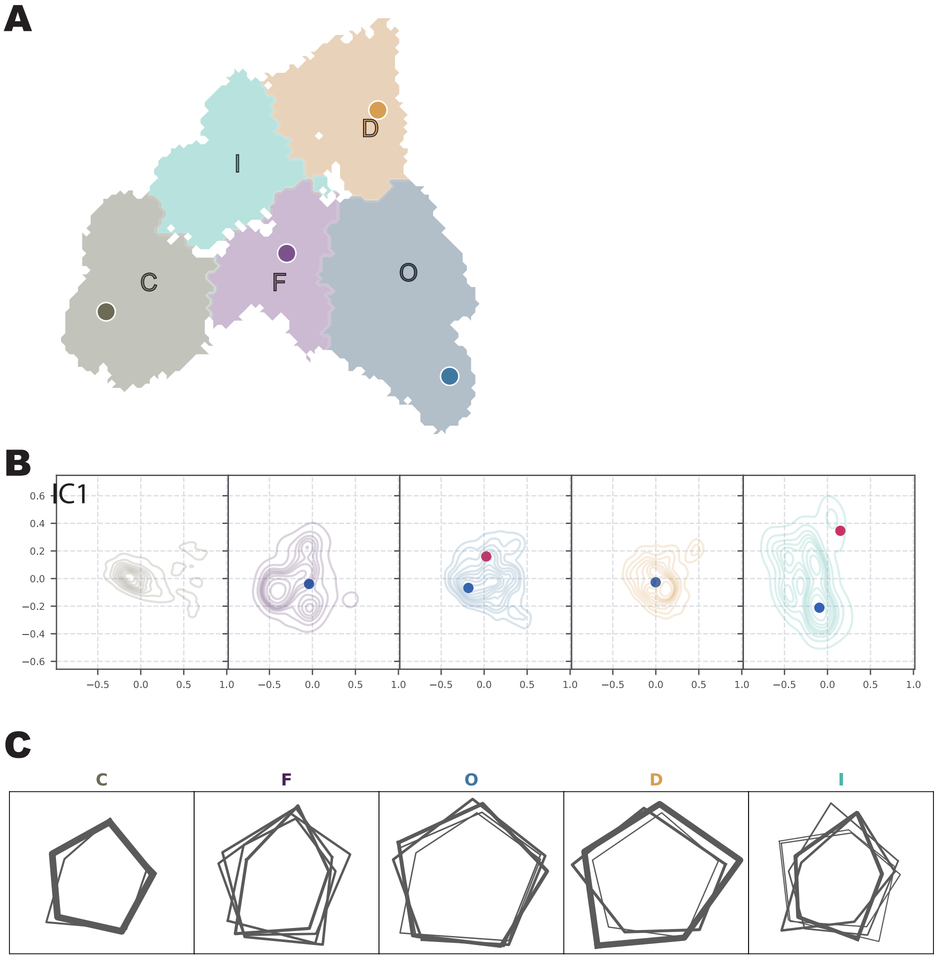

Supplementary Figure 11 MSM mapping and asymmetric analysis.(**A**) Mapping of a coarse-grained MSM with five macrostates onto IC1-IC2 coordinates in the CHOL system. Four structural models are projected onto the plot. (**B**) Symmetry-aware multidimensional scaling (MDS) projected sub Independent Components (IC1) onto the MDS1-MDS2 space. The zero point represents perfect symmetry with mean value, while a shifted distribution suggests sampling of asymmetric conformations. Representative snapshots in the free-energy basins are shown as dots (**C**) Representative snapshots of the $9'$ C_α pentagon captured in each macrostate at each local free energy minimum. The thickness of each pentagon indicates the relative population of that conformation within the corresponding macrostate.

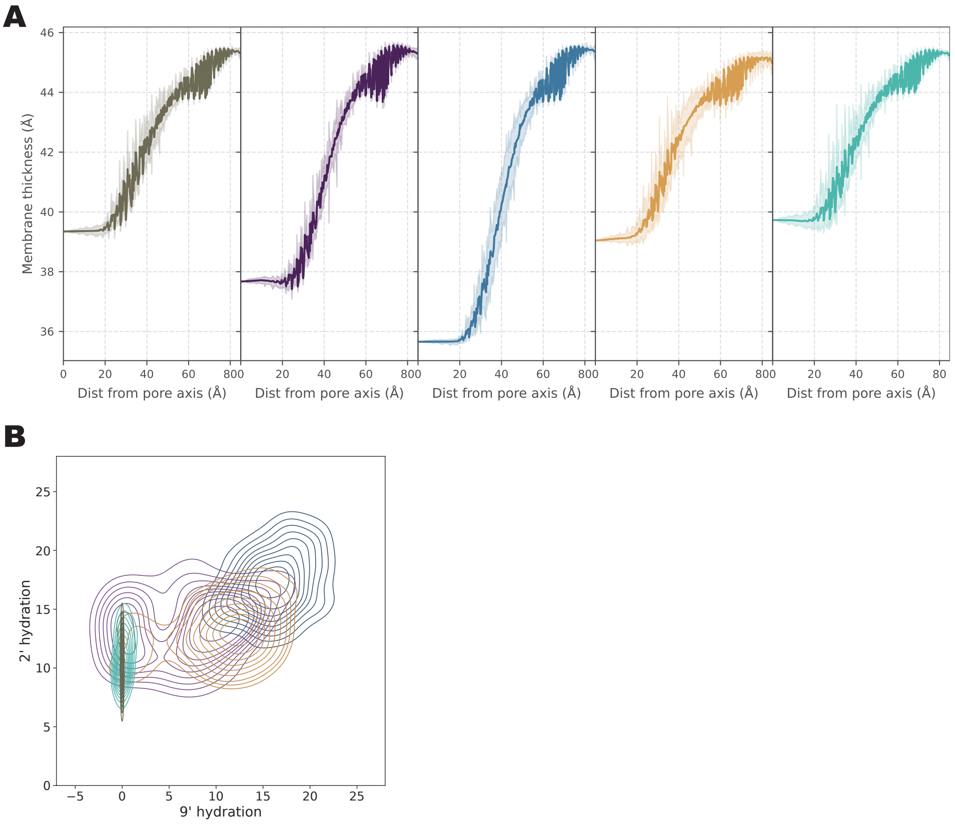

Supplementary Figure 12 Membrane compression and pore hydration profile in the CHOL system. (**A**) Membrane thickness profiles in each macrostate, obtained by radially averaging the membrane thickness centered at the pore axis. (**B**) Histogram of the number of water molecules around the 9′ residue versus the 2′ residue. This analysis was performed within a cylindrical region centered at each residue, extending ±2 Å along the pore axis.

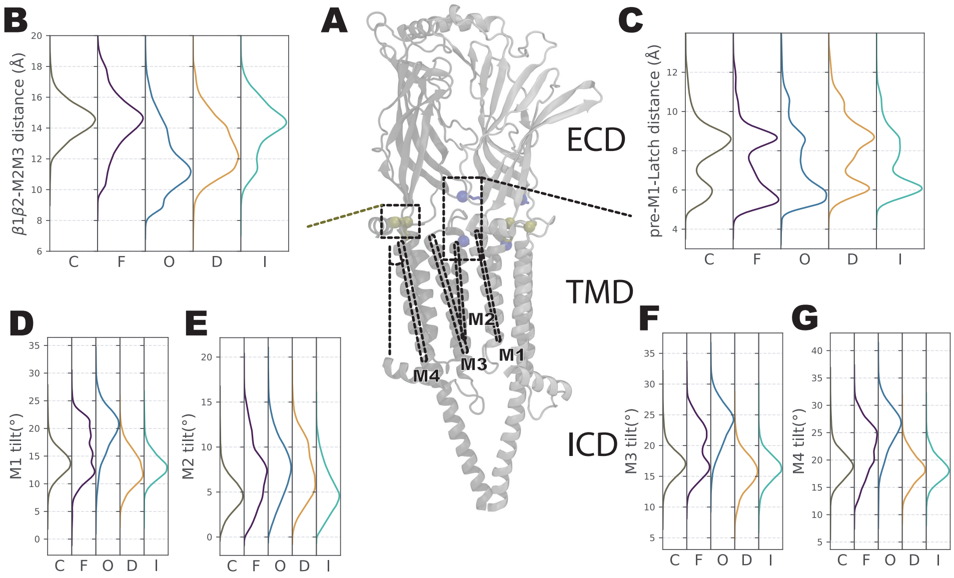

Supplementary Figure 13 Sequential conformational changes of coupling region and transmembrane domain in the CHOL system. (**A**) Visualization of two consecutive subunits, highlighting the contact between the pre-M1 loop and latch helix (shown in tan) and the contact between the β1-β2 loop and M2-M3 loop (shown in blue). The M1, M2, M3, and M4 helices are labeled with dashed lines. (**B**) Histogram showing the distribution of contacts between the pre-M1 loop and latch helix for each macrostate. (**C**) Histogram showing the distribution of contacts between the β1-β2 loop and M2-M3 loop for each macrostate. (**D, E, F, G**) Histograms showing the distribution of tilt angles for the M1, M2, M3, and M4 helices, respectively, in each macrostate. All distributions are weighted by the MSM weights.

Supplementary Note: Symmetric Toy model

Synthetic timeseries data generated from a given Markovian transition matrix between global states. Eight for a dimer with three sub-states; another eight for a trimer with two sub-states; 16 for a tetramer with two sub-states; In total six features comprised three observables in each subsystem. Conventional time-lagged independent component analysis (TICA) resolves all timescales while SymTICA (correctly) only resolves the degenerate model for the dynamics.

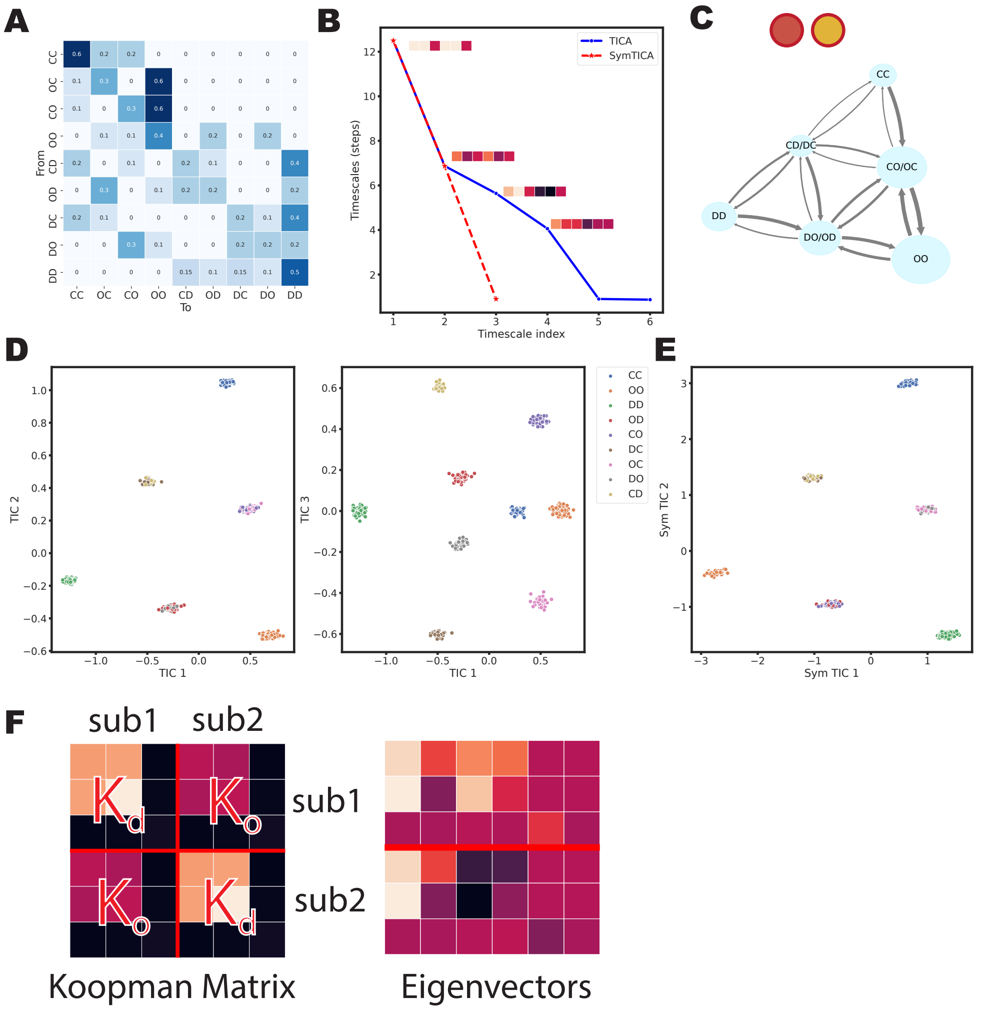

Supplementary Figure 14 Three-substate dimer toy model. (**A**) Transition probability matrix between all global states that comprise ordered subsystem states. (**B**) Resolved timescales by TICA and SymTICA. The schematic representation of projection vectors for each dimension is shown. SymTICA only resolves projections that are identical for each subsystem. (**C**) The degenerate Markov state diagram in which intermediate states are lumped together. (**D**) Data projected onto TIC 1-2-3 space by TICA, colored by their true global state. (**E**) Data projected onto Sym TIC 1-2 space by SymTICA. (**F**) Koopman matrix and eigenvectors for the full TICA decomposition. Koopman matrix can be separated into four blocks with the diagonal blocks being identical.

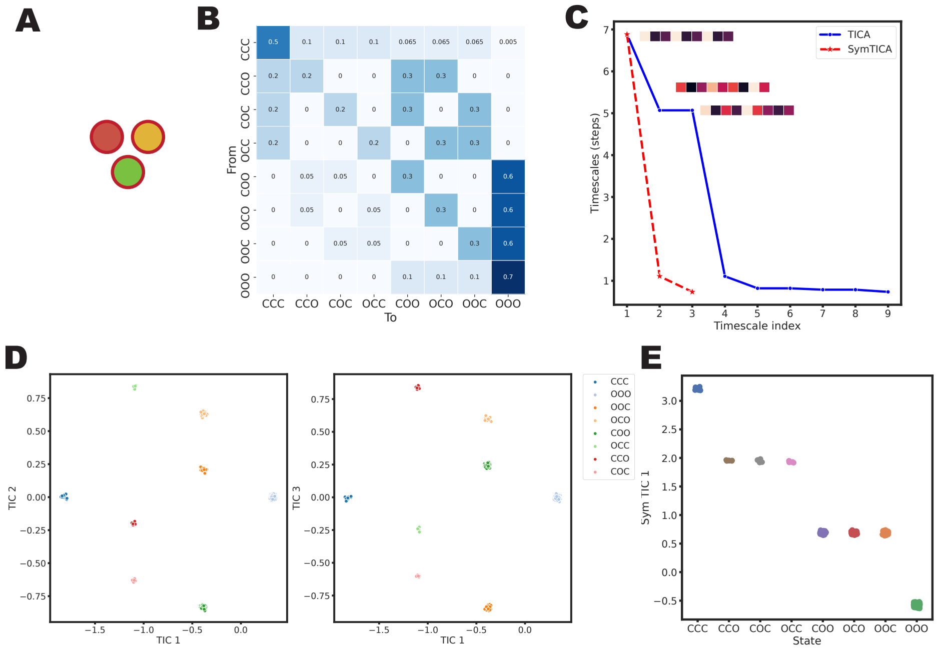

Supplementary Figure 15 Two-substate trimer toy model. (**A**) Schematic image of a trimer toy model. (**B**) Transition probability matrix between all global states that comprise ordered subsystem states. (**C**) Timescales resolved by TICA and SymTICA. The schematic representation of projection vectors for each dimension is shown. (**D**) Data projected onto TIC 1-2-3 space by TICA, colored by their true global state. (**E**) Data projected onto Sym TIC 1 space by SymTICA.

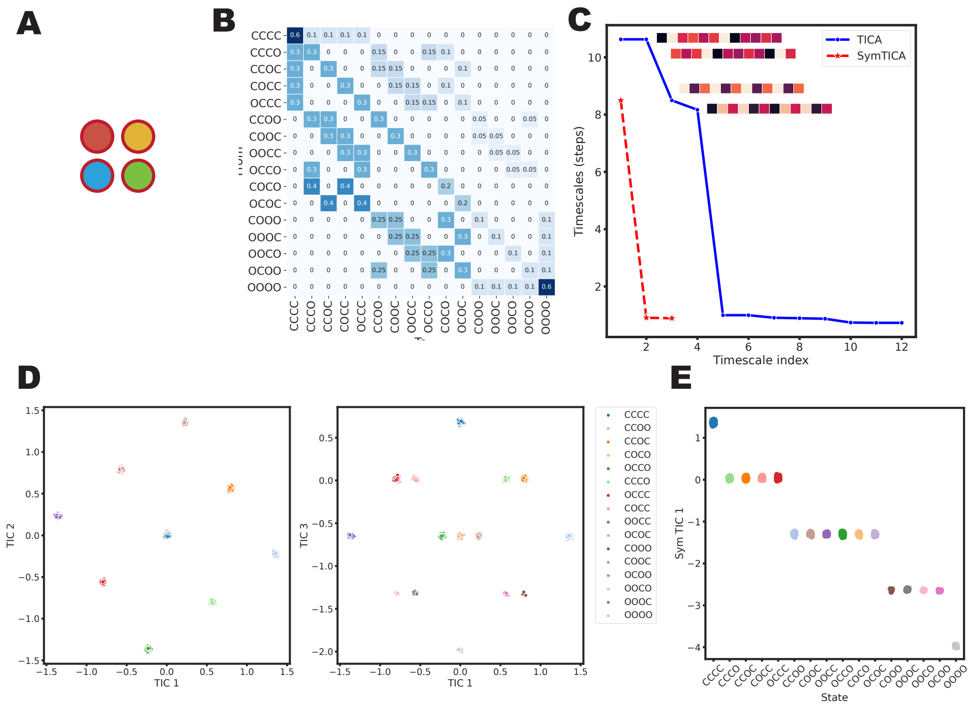

Supplementary Figure 16 Two-substate tetramer toy model (**A**) Schematic image of a tetramer toy model. (**B**) Transition probability matrix between all global states that comprise ordered subsystem states. (**C**) Timescales resolved by TICA and SymTICA. The schematic representation of projection vectors for each dimension is shown. (**D**) Data projected onto TIC 1-2-3 space by TICA, colored by their true global state. (**E**) Data projected onto Sym TIC 1 space by SymTICA.
