# Supplementary material for: Symmetry-adapted Markov state models of closing, opening, and desensitizing in α 7 nicotinic acetylcholine receptors
Source: Nat Commun. 2024 Oct 18;15:9022. doi: 10.1038/s41467-024-53170-z (PMC11489734; doi:10.1038/s41467-024-53170-z)
Supplement: Supplementary file 3 — Description of Additional Supplementary Files [file 41467_2024_53170_MOESM3_ESM.pdf]

Description of Additional Supplementary Files

File Name: Supplementary Movie 1

Description: Simulated 1-ms trajectory from an α7 MSM.

Visualization of α7-nAChR gating dynamics over 1 ms, based on assembling a trajectory from a simulation of the MSM kinetics. The highlighted circle indicates the current macrostate in tICA space, while the molecular representation visualizes a corresponding trajectory underlying the MSM (colored according to the macrostate), and the yellow hexagon indicates the projection of the current microstate snapshot onto tICA space.
