# Supplementary material for: Symmetry-adapted Markov state models of closing, opening, and desensitizing in α 7 nicotinic acetylcholine receptors
Source: Nat Commun. 2024 Oct 18;15:9022. doi: 10.1038/s41467-024-53170-z (PMC11489734; doi:10.1038/s41467-024-53170-z)
Supplement: Supplementary file 5 — Reporting Summary [file 41467_2024_53170_MOESM5_ESM.pdf]

nature portfolio

Corresponding author(s): Erik Lindahl

Last updated by author(s): May 6, 2024

Reporting Summary

Nature Portfolio wishes to improve the reproducibility of the work that we publish. This form provides structure for consistency and transparency in reporting. For further information on Nature Portfolio policies, see our Editorial Policies and the Editorial Policy Checklist.

Statistics

For all statistical analyses, confirm that the following items are present in the figure legend, table legend, main text, or Methods section.

n/a	Confirmed	
☐	☒	The exact sample size (*n*) for each experimental group/condition, given as a discrete number and unit of measurement
☐	☒	A statement on whether measurements were taken from distinct samples or whether the same sample was measured repeatedly
☒	☐	The statistical test(s) used AND whether they are one- or two-sided<br>*Only common tests should be described solely by name; describe more complex techniques in the Methods section.*
☒	☐	A description of all covariates tested
☐	☒	A description of any assumptions or corrections, such as tests of normality and adjustment for multiple comparisons
☐	☒	A full description of the statistical parameters including central tendency (e.g. means) or other basic estimates (e.g. regression coefficient) AND variation (e.g. standard deviation) or associated estimates of uncertainty (e.g. confidence intervals)
☒	☐	For null hypothesis testing, the test statistic (e.g. *F*, *t*, *r*) with confidence intervals, effect sizes, degrees of freedom and *P* value noted<br>*Give P values as exact values whenever suitable.*
☐	☒	For Bayesian analysis, information on the choice of priors and Markov chain Monte Carlo settings
☒	☐	For hierarchical and complex designs, identification of the appropriate level for tests and full reporting of outcomes
☐	☒	Estimates of effect sizes (e.g. Cohen's *d*, Pearson's *r*), indicating how they were calculated

Our web collection on statistics for biologists contains articles on many of the points above.

Software and code

Policy information about availability of computer code

Data collection	GROMACS-2021, Climber-v1.0
Data analysis	MDAnalysis-v2.8.0, ENPMDA (https://github.com/yuxuanzhuang/ENPMDA); custom code at https://github.com/yuxuanzhuang/msm_a7_nachrs (56f2b0d) and https://github.com/yuxuanzhuang/sym_msm (cb2a3b5) also available at DOI 10.5281/zenodo.8424867

For manuscripts utilizing custom algorithms or software that are central to the research but not yet described in published literature, software must be made available to editors and reviewers. We strongly encourage code deposition in a community repository (e.g. GitHub). See the Nature Portfolio guidelines for submitting code & software for further information.

Data

Policy information about availability of data

All manuscripts must include a data availability statement. This statement should provide the following information, where applicable:

- Accession codes, unique identifiers, or web links for publicly available datasets
- A description of any restrictions on data availability
- For clinical datasets or third party data, please ensure that the statement adheres to our policy

Representative snapshots from each microstate and final MSM models have been deposited on Zenodo at https://zenodo.org/records/11117001. The source data underlying Figures 1D-E, 2B, 2D, 2E, 3A-B, 4B-G, 5A-C, 6B, 6D, and Supplementary Figures are provided as a Source Data file. Structures with accession codes 7KOX, 7KOO, and 7KOQ were used as starting models. The scripts to generate figures can be found on GitHub at https://github.com/

yuxuanzhuang/msm_a7_nachrs. The implementation of SymTICA can be found at https://github.com/yuxuanzhuang/sym msm. Code and scripts that reproduce the results are also available on Zenodo at https://zenodo.org/records/11117001 (DOI 10.5281/zenodo.8424867).

Research involving human participants, their data, or biological material

Policy information about studies with human participants or human data. See also policy information about sex, gender (identity/presentation), and sexual orientation and race, ethnicity and racism.

Reporting on sex and gender	This research did not involve human participants, their data, or biological material.
Reporting on race, ethnicity, or other socially relevant groupings	This research did not involve human participants, their data, or biological material.
Population characteristics	This research did not involve human participants, their data, or biological material.
Recruitment	This research did not involve human participants, their data, or biological material.
Ethics oversight	This research did not involve human participants, their data, or biological material.

Note that full information on the approval of the study protocol must also be provided in the manuscript.

Field-specific reporting

Please select the one below that is the best fit for your research. If you are not sure, read the appropriate sections before making your selection.

☒ Life sciences ☐ Behavioural & social sciences ☐ Ecological, evolutionary & environmental sciences

For a reference copy of the document with all sections, see nature.com/documents/nr-reporting-summary-flat.pdf

Life sciences study design

All studies must disclose on these points even when the disclosure is negative.

Sample size	No statistical method was used to predetermine sample sizes. MD simulations were assessed on the basis of individual frames sampled on the order of nanoseconds, the scale of which were determined based on convergence of variables (e.g. Markov state model timescales) as documented in Supplementary Information.
Data exclusions	No data were excluded.
Replication	Following conventions of the field, individual simulations were not replicated; rather, degenerate states sampled from 150 different initial seeds were identified by Markov state modeling.
Randomization	As parallel simulations were performed on indistinguishable compute hardware, no randomization was performed nor covariates explicitly controlled.
Blinding	According to standard practices in molecular dynamics simulations, no blinding was deemed necessary, as the measured variables are not substantially subject subject to investigator influence.

Reporting for specific materials, systems and methods

We require information from authors about some types of materials, experimental systems and methods used in many studies. Here, indicate whether each material, system or method listed is relevant to your study. If you are not sure if a list item applies to your research, read the appropriate section before selecting a response.

Materials & experimental systems

n/a	Involved in the study	
☒	☐	Antibodies
☒	☐	Eukaryotic cell lines
☒	☐	Palaeontology and archaeology
☒	☐	Animals and other organisms
☒	☐	Clinical data
☒	☐	Dual use research of concern
☒	☐	Plants

Methods

n/a	Involved in the study	
☒	☐	ChIP-seq
☒	☐	Flow cytometry
☒	☐	MRI-based neuroimaging

Plants

Seed stocks	*Report on the source of all seed stocks or other plant material used. If applicable, state the seed stock centre and catalogue number. If plant specimens were collected from the field, describe the collection location, date and sampling procedures.*
Novel plant genotypes	*Describe the methods by which all novel plant genotypes were produced. This includes those generated by transgenic approaches, gene editing, chemical/radiation-based mutagenesis and hybridization. For transgenic lines, describe the transformation method, the number of independent lines analyzed and the generation upon which experiments were performed. For gene-edited lines, describe the editor used, the endogenous sequence targeted for editing, the targeting guide RNA sequence (if applicable) and how the editor was applied.*
Authentication	*Describe any authentication procedures for each seed stock used or novel genotype generated. Describe any experiments used to assess the effect of a mutation and, where applicable, how potential secondary effects (e.g. second site T-DNA insertions, mosiacism, off-target gene editing) were examined.*
